# Effects of the nursing practice environment, slow nursing, and living a calling on geriatric nursing stress among general hospital nurses in South Korea: a cross-sectional descriptive study

**DOI:** 10.7586/jkbns.25.074

**Published:** 2026-02-24

**Authors:** Chung Hee Woo

**Affiliations:** College of Nursing, Konyang University, Deajeon, Korea

**Keywords:** Geriatric nursing, Occupational stress, Vocational guidance, Work conditions

## Abstract

**Purpose:**

This study examined factors influencing geriatric nursing stress among general hospital nurses, with a focus on the nursing practice environment, slow nursing, and living a calling.

**Methods:**

This descriptive correlational study surveyed 176 nurses with recent experience in geriatric nursing using an online questionnaire administered in September 2025. Data were analyzed using descriptive statistics, the independent *t*-test, one-way analysis of variance, Pearson correlation coefficients, and multiple regression analysis with SPSS version 25.0.

**Results:**

Geriatric nursing stress was negatively correlated with living a calling (r = −.19, *p* = .013) and the nursing practice environment (r = −.29, *p* < .001), but positively correlated with slow nursing (r = .29, *p* < .001). Among the subdomains, staffing and resource adequacy within the nursing practice environment (r = −.37, *p* < .001) and comfort provision within slow nursing (r = .32, *p* < .001) showed the strongest associations with geriatric nursing stress. In multiple regression analysis, staffing and resource adequacy (β = −.24, *p* = .011) and comfort provision (β = .23, *p* = .009) were significant predictors of geriatric nursing stress, collectively explaining 27% of the variance.

**Conclusion:**

Improving staffing and resource adequacy within the nursing practice environment may help reduce geriatric nursing stress. Because comfort provision was identified as a positive predictor of stress, organizational strategies are needed to support nurses in delivering comfort-focused care without increasing stress. Reducing geriatric nursing stress is critical for promoting nurse well-being and ensuring organizational sustainability.

## INTRODUCTION

### 1. Background

As of June 2025, South Korea has become a super-aged society, with 10,558,842 persons aged 65 years or older accounting for 20.6% of the total population [1]. As of 2023, older adults had 2.2 physician-diagnosed chronic diseases, on average. Further, 5.2% of this age group had been hospitalized (excluding long-term care hospitals) during the previous year, with a mean length of stay of 17.3 days [2]. In 2022, national health insurance expenditures surpassed 100 trillion Korean won (KRW), with those aged 65 and older accounting for 43.1% of the total [3]. Population aging is a global social phenomenon, and the United Nations (UN) has urged member states to develop healthcare systems that meet the medical needs of older adults [4].

Older adult inpatients who are cared for by clinical nurses typically present with complex health problems that require specialized care [5]. Beyond acute admission issues, the age-related decline of physical and cognitive functions mean that older adults require more direct nursing care than other age groups. They are also relatively vulnerable to safety incidents such as falls, which necessitates repeated education for both patients and caregivers, even in daily-living domains beyond treatment and procedures [5-7]. Even among nurses with adequate geriatric nursing knowledge and attitudes, such circumstances can lead to burnout, perceived nursing burden, and stress [8,9]. Hospital nurses’ job-related stress contributes not only to individual outcomes such as physical health problems, burnout, anxiety, and job dissatisfaction, but also to organizational risks by lowering performance and engagement and thereby threatening organizational goals [10]. Previous work identified geriatric nursing stress as a key variable affecting the quality of geriatric nursing performance [11]. Building on this knowledge, parallels with the consequences of general job stress can be reasonably inferred. Nevertheless, to provide nursing services commensurate with the rising demand for geriatric care in an aging era, a more specific and empirical investigation of stress related to geriatric nursing is needed.

Geriatric nursing stress is often more intense in care domains that combine the characteristics of older patients (e.g., multimorbidity and geriatric syndromes) with nursing tasks, as well as in treatment-related decision-making processes that require close interactions—not only with the patient, but also with caregivers [12]. In general, work-related stress is affected by factors at the organizational and environmental levels, along with individual-level factors [10]. However, in geriatric nursing, the stress from caregivers and patients exceeds work-related stress [9]. Previous studies have therefore largely focused on individual-level variables, such as emotional labor and communication ability [12] or empathy [13]. Because interactions with older adults, whose response times in daily life and in coping with illness may slow due to declines in physical function and neural conduction velocity [14], constitute work-related stressors, we determined that an examination of organizational and environmental factors is also necessary.

The nursing practice environment refers to organizational characteristics as perceived by nurses, including the physical environment and organizational and policy elements that shape task execution and working methods, as well as interactions among organizational members [15]. Unstable schedules (night and rotating shifts), staffing shortages and heavy workloads, role ambiguity or conflict, and high assigned responsibility with limited rewards or promotion opportunities exacerbate nurses’ job stress [10]. While geriatric nursing stress may be understood in a similar context, clarifying the degree to which these factors relate specifically to geriatric nursing stress is important, to ensure that appropriate care aligns with patient characteristics, as counseled by the UN.

Slow nursing is a concept that has developed by adapting and synthesizing the social context of “slow” (in terms of slow food, art, city, and living) to clinical nursing practice [16]. Slow nursing enables nurses to provide observation and comfort tailored to each patient’s individual needs, without being constrained by time, thereby promoting qualitative interactions with patients [17]. Slow nursing is especially pertinent in geriatric care because older adults often cannot keep pace with routine nursing that is provided in the same manner as for general adult patients [18,19]. Domestic studies have therefore primarily examined slow nursing in long-term care hospitals, where the proportion of older adult inpatients is high [18–20]. The slow nursing performance differed clearly according to the hospital size, but it also varied depending on individual nurse characteristics across each hospital size [19]. Therefore, slow nursing can be regarded as a variable that reflects both individual and organizational dimensions. Slow nursing is associated with geriatric nursing stress among long-term care hospital nurses and can be diminished by geriatric nursing stress itself [18]. Because older adults increasingly use general hospitals for acute conditions, in addition to long-term care services [3], extending this line of inquiry across diverse hospital settings is meaningful. In productivity-oriented hospital environments, the application of slow nursing may differ [19], with corresponding implications for geriatric nursing stress.

When nurses encounter tasks or job-related stressors that cannot be explained solely by personal interest or aptitude, mobilizing positive affect [21] or re-articulating the meaning and purpose of their work may help them to cope [22]. In its modern sense, the definition of “calling” has evolved into a work-related construct that reflects an attitude of pursuing meaning and goals within one’s work while striving to contribute to the greater social and ethical good [23]. Duffy et al. [24]’s work on Calling Theory also emphasizes the role of calling. Calling can be linked to positive outcomes such as job satisfaction and performance. Likewise, it can yield negative outcomes, such as burnout or exploitation, thereby reflecting its dual nature [24]. “Living a calling”, which goes beyond the mere awareness of having a calling, captures the actual enactment of a calling in one’s work and life, and should be empirically investigated [25]. Living a calling has been shown to contribute positively to the perception of nursing as a “decent job,” even when job security is threatened [26]. Although there is a dearth of empirical studies that directly examine the concept of living a calling and geriatric nursing stress, we posit that nurses’ orientation toward connecting the meaning and goals of their work to the public good may buffer geriatric nursing stress.

Accordingly, this study aims to identify how the nursing practice environment, slow nursing, and living a calling affect geriatric nursing stress among general hospital nurses, and thereby provide foundational data to inform resources and strategies for managing geriatric nursing stress in clinical practice.

### 2. Study aim

The purpose of this study is to identify factors that influence geriatric nursing stress among general hospital nurses. The specific aims are as follows. First, we aim to determine participants’ general characteristics and their levels of geriatric nursing stress, nursing practice environment, slow nursing, and living a calling. Second, we examine differences in levels of geriatric nursing stress according to participants’ general characteristics. Third, we identify correlations of the nursing practice environment, slow nursing, and living a calling with geriatric nursing stress. Finally, we determine the effects of the nursing practice environment, slow nursing, and living a calling on geriatric nursing stress.

## METHODS

### 1. Study design

This descriptive correlational study examined the effects of nursing practice environment, slow nursing, and living a calling on geriatric nursing stress among general hospital nurses.

### 2. Participants

Participants were registered nurses with at least six months of clinical experience in general hospitals with 100 or more beds [27], and who had provided nursing care to older adults within the previous month. Using G*Power 3.1.9.4 with a two-sided α of .05, a power (1-β) of .90, a medium effect size of 0.15 [28], and sixteen predictors (general characteristics, independent variables, and their subdomains) for multiple regression, the required sample size was 175. Assuming a dropout rate of approximately 10%, the target sample size was 194, and data from 176 nurses were included in the final analysis.

### 3. Instruments

The questionnaire included 80 items covering five domains: general characteristics (5 items), geriatric nursing stress (17 items), nursing practice environment (29 items), slow nursing (23 items), and living a calling (6 items).

#### 1) General characteristics

Following previous studies, participants’ general characteristics included five items: sex, age, education, clinical experience, and participation in geriatric nursing-related education.

#### 2) Geriatric nursing stress

Geriatric nursing stress was measured using the Nurse Stress Scale developed by Kim and Gu [29], which was modified to assess geriatric nursing stress [9] and subsequently adapted for nurses in general hospitals [11]. The instrument comprises three subdomains: work-related stress (five items), patient-related stress (six items), and caregiver-related stress (six items), for a total of 17 items. Each item is rated on a 4-point Likert scale ranging from 1 (“strongly disagree”) to 4 (“strongly agree”), with higher scores indicating higher geriatric nursing stress. Cronbach’s α ranged from .66~.83 in Choi and Lee [9] and .73~.80 in Choi and Yoo [11]. In the present study, the Cronbach’s α was .84 for the total scale, while for the sub-items of work-related, patient-related, and caregiver-related stress, they were .77, .84, and .83, respectively.

#### 3) Nursing practice environment

The nursing practice environment variable was measured using the Korean version of the Practice Environment Scale of the Nursing Work Index. This instrument was originally developed by Lake [30] and translated by Cho et al. [31]. In the scale, the 29 items are grouped into five subdomains: nurse participation in hospital affairs (nine items), nursing foundations for quality of care (nine items), nurse manager characteristics (ability, leadership, and support; four items), staffing and resources adequacy (four items), and collegial nurse–physician relations (three items). Items are rated on a 4-point Likert scale ranging from 1 (“strongly disagree”) to 4 (“strongly agree”), with higher scores indicating a better practice environment. The Cronbach’s α was .82 for the original instrument [30], .93 in Cho et al. [31], and .94 in the present study.

#### 4) Slow nursing

Slow nursing was assessed using the instrument developed and validated by Woo [32] for nurses working in long-term care hospitals, which has also been applied in studies involving nurses from tertiary general hospitals, general hospitals, and long-term care hospitals [19]. This reflects the notion that ‘slow nursing’ should serve as a way of practicing the nursing profession across all care settings [16]. The instrument comprises five subdomains: pacing to the patient’s tempo (seven items), observation and attentiveness (seven items), comfort provision (three items), respect (three items), and imbuing life with value (three items), for a total of 23 items. Each item is rated on a 4-point Likert scale ranging from 1 (“strongly disagree”) to 4 (“strongly agree”), with higher scores indicating a higher level of slow nursing. The Cronbach’s α was .86 at development [32] and .85 in the present study.

#### 5) Living a calling

Living a calling was measured using the Living a Calling Scale–Korean, developed by Duffy et al. [33] and validated by Ahn and Shin [25]. The scale has six items rated on a 7-point Likert scale ranging from 1 (“strongly disagree”) to 7 (“strongly agree”), with higher scores indicating a stronger perception of living one’s calling. In the original development study [33], each item included an eighth response option, “Not applicable—I do not have a calling,” scored as 8. While it was decided that responses of 8 will be excluded from analysis, none were excluded from the present study. The Cronbach’s α was .94 in the development study [33], .95 in Ahn and Shin [25], and .94 in the present study.

### 4. Data collection

Data were collected from September 11 to 30, 2025. Convenience sampling was conducted with the assistance of research assistants who had access to online communities in the nursing departments of general hospitals located in Seoul, Gyeonggi Province, and the Chungcheong region. Nurses who wished to participate accessed the survey link via a QR code. The survey’s landing page was used to screen for eligibility by nurses confirming their employment for at least six months at a general hospital and their experience in providing nursing care to older adults within the past month. Those who met the inclusion criteria gained access to the study information sheet and voluntarily selected “agree” on the electronic consent form, after which they were directed to the questionnaire. The survey required approximately 10 minutes to complete, and a small token of appreciation was provided for participation. The target sample size was 194, but a total of 176 nurses participated. None were excluded from analysis.

### 5. Data analysis

Data were analyzed using SPSS version 25.0 (IBM Corp., Armonk, NY, USA), as follows: descriptive statistics were computed for participants’ general characteristics, geriatric nursing stress, nursing practice environment, slow nursing, and living a calling. Differences in geriatric nursing stress according to general characteristics were examined using independent *t*-tests and one-way analysis of variance, with Scheffé’s post hoc test. Correlations between geriatric nursing stress, nursing practice environment, slow nursing, and living a calling were calculated using Pearson correlation coefficients. Factors influencing geriatric nursing stress were identified using a multiple regression analysis with the enter method.

### 6. Ethical considerations

This study was approved by the Institutional Review Board of Konyang University (No. KYU-2025-08-004). Prior to completing the survey, participants were informed about the study purpose, their right to refuse or withdraw, and personal information protection. Electronic written consent was obtained: the survey platform clearly presented “agree/disagree” options after the participants had sufficient time to review the information sheet. Selecting “agree” opened the questionnaire, whereas selecting “disagree” automatically closed the survey and terminated participation. The participants were informed that they could withdraw at any time without penalty, and that upon withdrawal, all personal information and responses would be discarded. The information sheet also described measures implemented to ensure anonymity, confidentiality through coding, and personal information protection.

## RESULTS

### 1. General characteristics of participants

In the final sample, 20 (11.4%) of the participants were men and 156 (88.6%) were women. The mean age was 31.23 ± 5.17 years; 61 participants (34.7%) were aged 20~29, 107 (60.8%) 30~39, and 8 (4.5%) were aged 40 years or older. Regarding educational attainment, 19 (10.8%) participants held an associate degree, 117 (66.5%) a bachelor’s degree, and 40 (22.7%) had graduate-level education or higher. In terms of clinical experience, 30 (17.0%) participants had 3 years or less, 62 (35.2%) had 4~6 years, 51 (29.0%) 7~10 years, and 33 (18.8%) had 11 years or more. Regarding geriatric nursing education, most respondents (n = 126, 71.6%) reported having completed a university course and/or in-service continuing education, while the other 50 (28.4%) reported having no such experience (Table 1).

### 2. Levels of geriatric nursing stress, nursing practice environment, slow nursing, and living a calling

As reported in Table 2, the mean geriatric nursing stress score was 3.09 ± 0.44. By subdomain, caregiver-related stress (3.31 ± 0.56) was highest, followed by patient-related stress (3.05 ± 0.63) and work-related stress (2.89 ± 0.51). The mean score for the nursing practice environment was 2.32 ± 0.50; subdomain means were nurse participation in hospital affairs (2.66 ± 0.50), collegial nurse–physician relations (2.55 ± 0.64), nurse manager characteristics (2.32 ± 0.63), nursing foundations for quality of care (2.05 ± 0.56), and staffing and resources adequacy (2.04 ± 0.71). The average score for slow nursing was 2.91 ± 0.41; the subdomain means were comfort provision (3.28 ± 0.48), respect (3.26 ± 0.56), pacing to the patient’s tempo (2.90 ± 0.44), observation and attentiveness (2.88 ± 0.45), and imbuing life with value (2.27 ± 0.72). Lastly, the average score of living a calling was 4.77 ± 1.08. Normality was assessed using absolute skewness and kurtosis; absolute kurtosis ranged from 0.13 to 1.00 (< 10) and absolute skewness from 0.01 to 0.94 (< 3), indicating approximate normal distributions.

### 3. Differences in geriatric nursing stress by general characteristics

Geriatric nursing stress differed significantly by age (t = 3.16, *p* = .045). According to the post hoc comparisons, nurses aged 30~39 reported significantly higher geriatric nursing stress than those aged 20~29. No significant differences were found in terms of sex (t = −1.72, *p* = .088), education level (t = 0.06, *p* = .947), clinical experience (t = 1.84, *p* = .142), or presence/absence of geriatric-nursing education experience (t = −0.43, *p* = .671) (Table 3).

### 4. Associations of nursing practice environment, slow nursing, and living a calling with geriatric nursing stress

As shown in Table 4, geriatric nursing stress was significantly and negatively correlated with the nursing practice environment (r = −.29, *p* < .001) and living a calling (r = −.19, *p* = .013), but positively correlated with slow nursing (r = .29, *p* < .001). At the subdomain level, significant correlations with geriatric nursing stress were observed in three nursing practice environment subdomains: nursing foundations for quality of care (r = −.22, *p* = .004), nurse manager characteristics (r = −.32, *p* < .001), and staffing and resources adequacy (r = −.37, *p* < .001). Significant correlations were also observed for four slow nursing subdomains: pacing to the patient’s tempo, observation and attentiveness (r = .25, *p* = .001), comfort provision (r = .32, *p* < .001), and respect (r= .22, *p* = .004).

### 5. Factors influencing geriatric nursing stress

To clarify the predictors of geriatric nursing stress, we first conducted correlation analyses at the subdomain level and then performed simple regressions on using variables that were statistically significant. For the simple regressions, age, a categorical variable, was dummy-coded using nurses aged 30~39 as the reference group. Variables showing significance in the simple analyses (age: 20~29; nursing practice environment: nursing foundations for quality of care, nurse manager characteristics, staffing and resources adequacy; slow nursing: pacing to the patient’s tempo, observation and attentiveness, comfort provision, respect; and living a calling) were then entered as predictors in a multiple regression.

Assumption checks determined acceptable collinearity (tolerance = .36~.94; variance inflation factor = 1.06~2.79), independence of errors (Durbin–Watson = 2.01), and normality of residuals (Q–Q plot approximating the 45° line).

In the multiple regression, staffing and resources adequacy (β = −.24, *p* = .011) and comfort provision (β = .23, *p* = .009) emerged as significant predictors of geriatric nursing stress. The model explained 27% of the variance in geriatric nursing stress scores (Table 5).

## DISCUSSION

One strategy for enhancing sustainability in nursing within a super-aged society is to effectively manage the work burden associated with care for the growing population of older adults. Accordingly, this study examined how the nursing practice environment, slow nursing, and living a calling affect geriatric nursing stress among general hospital nurses, with the aim of providing foundational data for strategies to manage geriatric nursing stress.

The mean level of geriatric nursing stress among participants was slightly higher than that of nurses in a cancer specialty hospital [13], that of nurses in general hospitals in the Seoul–Gyeonggi area [5,9], and that of nurses at a regional university hospital [12]. Overall, their level is closest to that of nurses in a regional general hospital [11]. Despite differences in survey timing and setting, these findings imply that the stress level associated with geriatric nursing in clinical practice has not improved. By subdomain, caregiver-related stress was shown to be the highest (3.31), which is consistent with numerous existing studies [5,9,11,13]; this indicates that strategies to mitigate stress in geriatric care should prioritize caregiver-related factors. Because older adults face increased risks of safety incidents, in addition to disease-related issues [5], the direct nursing burden is substantial; nonetheless, measures that reduce caregiver-related stress are likely to be beneficial. In this regard, the active adoption of the comprehensive nursing care service—which was piloted in 2007 and gradually expanded in wards and institutions with high volumes of older patients [34]—may help alleviate nurses’ geriatric nursing stress.

Our analysis of participant characteristics revealed significant differences in geriatric nursing stress across age groups, with nurses in their 30s reporting higher stress than those in their 20s. Although not statistically significant, stress levels were recorded to be higher among nurses with ≥ 7 years of clinical experience, compared with those with ≤ 6 years, and slightly higher among those without geriatric-nursing education experience. Our results differ from some studies in which stress was higher among nurses with geriatric-care training [5] or those that showed no consistent pattern by age, experience, or education [9,12]. To deepen our understanding of geriatric nursing stress among general hospital nurses, future studies should account for hospital size, regional hubs, and other contextual factors and include these characteristics as control variables where results have been inconsistent.

Our correlation analyses revealed that the nursing practice environment—specifically, nursing foundations for quality of care, nurse manager characteristics, and staffing and resources adequacy—as well as living a calling were negatively associated with geriatric nursing stress, whereas slow nursing and its subdomains (pacing to the patient’s tempo, observation and encouragement of engagement, comfort provision, and respect) were positively associated. Together with findings that the nursing practice environment is positively related to geriatric nursing practice in comprehensive nursing care wards [35] and geriatric nursing performance in tertiary hospitals [36], these results underscore the strong association between the practice environment and geriatric-care variables. They also correspond to evidence that nurses’ job stress across diverse settings [37,38], and all five subdomains of the nursing practice environment, are inversely related to burnout among general hospital nurses [39]. However, because few studies have examined subdomain-level associations (aside from Kim and Lee [39]), comparisons should be made with caution. Nurse stress is not only detrimental to physical and mental health at an individual level, it also undermines job satisfaction, organizational commitment, and nursing outcomes, ultimately increasing turnover intention [10]. Therefore, analyses that capture detailed features of the nursing practice environment are necessary to clarify geriatric nursing stress and develop effective countermeasures.

The inverse association between living a calling and geriatric nursing stress confirms existing work that identifies the former as a resource that supports positive work perceptions and satisfaction [24,26]. In contrast, the positive association between slow nursing and geriatric nursing stress in the current study diverges from findings in long-term care hospitals where the former was negatively associated with stress [18]. This discrepancy may reflect environmental differences: compared with long-term care settings in which the focus is on chronic geriatric conditions [37], general hospitals inevitably manage a higher proportion of acute and emergency cases. Although the practice of slow nursing may enhance the qualitative level of care, it might also have imposed an additional burden in environments constrained by limited staffing and time resources. Given that the levels of slow nursing can vary by hospital size [19] and organizational philosophy and values, even in long-term care settings [18,37], caution is required for interpretation. Nevertheless, if nursing is to pursue high-quality care, empirical foundations that foster slow nursing, particularly its emphasis on alignment with the patient’s tempo, should be developed across diverse nursing groups. Moreover, although living a calling has been found to positively influence individuals’ perceptions of their work and life [25] and contributing to the perception of nursing as a decent job even under employment insecurity [26], it is noteworthy that its association with geriatric nursing stress was relatively weaker than that of other variables.

To identify factors influencing geriatric nursing stress, variables that were significant in the preliminary analyses were first tested, using simple regressions. Next, significant variables were entered into a multiple regression. Only the subdomain of staffing and resources adequacy within the nursing practice environment and that of comfort provision within slow nursing significantly predicted geriatric nursing stress; this pattern is consistent with existing evidence showing that greater staffing and resources adequacy reduce burnout among general hospital nurses [39] and that higher geriatric nursing stress is associated with lower levels of slow nursing among long-term care nurses [18]. In contrast to one previous report [39], nurse manager characteristics were not significant in our model.

Given that the nursing practice environment encompasses factors ranging from physical resources supporting quality care to institutional policies [40], system-wide improvements may be the most effective means to alleviate nurse burnout and work-related stress [41]. If resources must be allocated sequentially, nurse staffing and resources support should be prioritized. Moreover, inadequate work environments that elicit stress directly and indirectly affect intention to remain and work performance [40]; thus, improving the nursing practice environment could both reduce individual stress and enhance organizational sustainability. Slow nursing is aligned with critiques of healthcare that is overly focused on speed and efficiency [17]. Given the pervasive nursing practice environment is a strategy that can both reduce individual stress and enhance high-quality care that satisfies older adults’ medical needs [16]. When care proceeds at a pace that enables patient comfort, patients perceive it as good care, and therefore trust nurses more readily [42]. However, our findings suggest that comfort provision, which is an essential nursing activity for older adults with atypical and complex geriatric conditions, was a major source of stress, likely because nurses perceive comfort provision as indispensable, while facing heavy workloads amid insufficient staffing and support. As “slowness” signifies appropriate attentiveness rather than mere speed, such an approach may also support efforts to prevent frequent safety incidents among hospitalized older adults [43]. Thus, slow nursing may play a role in reducing safety incidents among older adults and easing nurses’ stress in geriatric care, and may therefore represent a long-term approach that potentially mitigates risks for older patients.

We anticipated that living a calling would play a meaningful role in situations where stress intensifies during the often-disfavored task of geriatric nursing [8,9]. However, compared with the nursing practice environment and slow nursing, this factor did not exert a statistically significant influence in our multivariable model. Although living a calling has been linked to job satisfaction, performance, and burnout [24] and to perceiving nursing as a decent job [26], our findings suggest that the stress experienced while delivering high-quality care that responds to the growing needs of older patients may not be adequately addressed by appealing to individual calling alone. Rather, efforts should focus on improving the nursing practice environment, so that care can be paced to the “slowness” inherent to older patients. Because the relative effects among the simultaneously entered variables cannot be ruled out, replication across diverse settings, populations, and variable sets is warranted.

This study examined how the nursing practice environment, slow nursing, and living a calling affect the stress of geriatric nursing in the context of a super-aged society. The key implication is that strategies to manage geriatric nursing stress should prioritize organizational-level interventions, rather than individual psychological traits. Specifically, ensuring adequate staffing and physical resources to enable comfort provision at a tempo appropriate to older adults—who inevitably require “slowness”—should be a first-order priority.

The limitations of this study are as follows. First, because participants were convenience-sampled from general hospitals with permitted access to an online community, the generalizability of the findings should be interpreted with caution. Second, feasibility constraints limited the number of variables included, which may have influenced the results. Third, although this study highlighted its biological nursing relevance by evaluating workload burden through the physiologically grounded concept of stress, it did not include physiological indicators due to reliance on self-reported measures. Finally, some variables that are central to nursing philosophy may not be easily valued within a market-economy logic.

## CONCLUSION

This descriptive correlational study identified factors influencing geriatric nursing stress among general hospital nurses. Staffing and physical resources within the nursing work environment and the comfort provision domain of slow nursing were identified as significant predictors of geriatric nursing stress. In the current super-aged era, understanding stress related to geriatric care and developing strategies to mitigate it are crucial. Above all, societal consensus and institutional support are needed to strengthen the nursing work environment so that nurses can provide comfort at the “slower” tempo inherently required by older adult patients.

Based on the study results, the following recommendations are suggested. First, future studies are encouraged to recruit participants from a more diverse range of hospital types and settings to enhance representativeness. Second, it is recommended that subsequent research incorporate a broader set of individual-level psychological factors to more comprehensively examine their interrelationships. Third, future research should include physiological indicators alongside self-reported measures to more rigorously assess the physiological nature of stress within nursing workloads. Finally, future studies are encouraged to empirically explore how the core values of nursing affect organizational sustainability.

## Figures and Tables

**Table 1.** Sociodemographic and Work-related Characteristics of the Participants (N = 176)

Variables	Categories	n (%)	M ± SD
Sex	Men	20 (11.4)	
	Women	156 (88.6)	
Age (years)	20~29	61 (34.7)	31.23 ± 5.17
	30~39	107 (60.8)	
	≥ 40	8 (4.5)	
Education level	Associate degree	19 (10.8)	
	Bachelor’s degree	117 (66.5)	
	Graduate degree	40 (22.7)	
Clinical experience (years)	≤ 3	30 (17.0)	
	4~6	62 (35.2)	
	7~10	51 (29.0)	
	≥ 11	33 (18.8)	
Geriatric nursing education (taking a university course or hospital program)	Yes	126 (71.6)	
	No	50 (28.4)	

M = Mean; SD = Standard deviation.

**Table 2.** Levels of Geriatric Nursing Stress, Nursing Practice Environment, Slow Nursing, and Living a Calling (N = 176)

Variables	M	SD	Min	Max	Skewness	Kurtosis
Geriatric nursing stress	3.09	0.44	1.65	4.00	−0.51	0.27
Work-related stress	2.89	0.51	1.40	4.00	−0.01	0.28
Patient-related stress	3.05	0.63	1.00	4.00	−0.63	0.43
Caregiver-related stress	3.31	0.56	1.50	4.00	−0.94	0.57
Nursing practice environment	2.32	0.50	1.00	3.57	0.10	−0.26
Nurse participation in hospital affairs	2.66	0.50	1.00	3.89	−0.46	0.66
Nursing foundations for quality of care	2.05	0.56	1.00	3.22	0.31	−0.76
Nurse manager characteristics	2.32	0.63	1.00	4.00	0.20	−0.32
Staffing and resources adequacy	2.04	0.71	1.00	3.75	0.32	−0.82
Collegial nurse-physician relations	2.55	0.64	1.00	4.00	−0.41	−0.32
Slow nursing	2.91	0.41	1.96	3.78	0.09	−0.25
Pacing to the patient's tempo	2.90	0.44	1.43	3.86	−0.54	0.18
Observation and attentiveness	2.88	0.45	1.43	4.00	0.09	0.13
Comfort Provision	3.28	0.48	2.00	4.00	−0.31	0.09
Respect	3.26	0.56	2.00	4.00	−0.51	−0.29
Imbuing life with value	2.27	0.72	1.00	4.00	0.24	−0.54
Living a calling	4.77	1.08	1.00	7.00	−0.66	1.00

M = Mean; SD = Standard deviation; Min = Minimum; Max = Maximum.

**Table 3.** Differences in Geriatric Nursing Stress According to General Characteristics (N = 176)

Variables	Categories	M ± SD	t or F	*p* (Scheff)
Sex	Men	2.94 ± 0.44	−1.72	.088
	Women	3.12 ± 0.44		
Age (years)	20~29^a^	2.98 ± 0.51	3.16	.045 (a<b)
	30~39^b^	3.16 ± 0.40		
	≥ 40^c^	3.15 ± 0.36		
Education level	Associate degree	3.07 ± 0.39	0.06	.947
	Bachelor’s degree	3.10 ± 0.47		
	Graduate degree	3.09 ± 0.44		
Clinical experience (years)	≤ 3	3.03 ± 0.52	1.84	.142
	4~6	3.02 ± 0.47		
	7~10	3.18 ± 0.32		
	≥ 11	3.17 ± 0.44		
Geriatric nursing education	Yes	3.08 ± 0.45	−0.43	.671
	None	3.12 ± 0.44		

M = Mean; SD = Standard deviation.

**Table 4.** Associations of Nursing Practice Environment, Slow Nursing, and Living a Calling with Geriatric Nursing Stress (N = 176)

Variables	Geriatric nursing stress	*p*
	r	
Nursing practice environment (total)	−.29	< .001
Nurse participation in hospital affairs	−.12	.125
Nursing foundations for quality of care	−.22	.004
Nurse manager characteristics	−.32	< .001
Staffing and resources adequacy	−.37	< .001
Collegial nurse-physician relations	−.13	.077
Slow nursing (total)	.29	< .001
Pacing to the patient's tempo	.25	.001
Observation and attentiveness	.25	.001
Comfort provision	.32	< .001
Respect	.22	.004
Imbuing life with value	.12	.123
Living a calling	−.19	.013

Sub-factors of nursing practice environment and slow nursing were treated as continuous variables.

**Table 5.** Factors Influencing Geriatric Nursing Stress (N = 176)

Predictors		B	SE	β	t	*p*
Constant		2.57	0.25		10.45	< .001
Age (years)	20~29 (ref = 30~39)	−0.10	0.06	−.11	−1.58	.117
Nursing practice environment	Nursing foundations for quality of care	0.06	0.08	.08	0.76	.450
	Nurse manager characteristics	−0.15	0.08	−.21	−1.95	.053
	Staffing and resources adequacy	−0.15	0.06	−.24	−2.57	.011
Slow nursing	Pacing to the patient's tempo	0.01	0.10	.01	0.08	.934
	Observation and attentiveness	0.09	0.09	.09	1.00	.321
	Comfort provision	0.22	0.08	.23	2.65	.009
	Respect	0.10	0.07	.13	1.39	.166
Living a calling		−0.05	0.04	−.12	−1.42	.159
R^2^ = .30, Adjusted R^2^ = .27, F = 8.24, *p* < .001						

SE = Standard error; ref = reference.

## Data Availability

Please contact the author for data availability.
